# Evaluation of agreement for radiographic lesions and risk for racing in thoroughbred yearling sale repository radiographs

**DOI:** 10.3389/fvets.2024.1430993

**Published:** 2024-10-03

**Authors:** Brodie J. Argue, Benjamin J. Ahern

**Affiliations:** School of Veterinary Science, University of Queensland, Gatton Campus, Gatton, QLD, Australia

**Keywords:** yearling, prepurchase assessment, thoroughbred horses, thoroughbred racehorse, radiogaphy

## Abstract

**Introduction:**

The veterinary yearling pre-sale repository radiographs have the potential to impact both vendor and prospective purchaser. The primary aim of this study was to determine inter-observer agreement for orthopaedic lesions identified in thoroughbred yearling repository radiographs. A secondary goal was to determine agreement when using a pre-defined risk rating classification.

**Methods:**

Three experienced specialist equine surgeons (2 ACVS & 1 FANZCVS) interpreted thirty repository radiographs once each for radiographic abnormalities. Each radiographic abnormality was given an associated risk assessment for future racing performance.

**Results:**

The use of a pre-defined risk rating grading scale resulted in good to excellent agreement as observers reached a consensus on risk ratings for lesions 97.1% of the time. The highest agreement was for the proximal interphalangeal joint and distal interphalangeal joint, with 99.9 and 98.2% agreement, respectively. The tarsal region was the joint with the highest disagreement with respect to pathology, with observers disagreeing 5.2% of the time.

**Discussion:**

This study found that experienced veterinary surgeons reliably agreed on the absence of lesions but showed disagreement on the specific risk rating of common lesions.

## Introduction

Thoroughbred yearling sale repositories play an essential role in the equine industry, serving as archives for radiographs of individual yearlings showcased at select thoroughbred sales. The interpretation of yearling repository radiographs often evokes controversy, given its potential for profound financial ramifications for vendors and prospective owners alike. Radiographic interpretation in the veterinary pre-purchase assessment is significantly influenced by various factors, including the published literature, clinical experience of veterinary surgeons and the diversity of their client base. Additionally, the presence or absence of pathology can affect the sale of a yearling. Despite the importance of radiography in highlighting radiographic abnormalities, the consistency and reliability of radiographic interpretation have received little attention in the literature.

During the assessment of repository radiographs, veterinary surgeons must make a judgement on the possible effect radiographic lesions will have on future racing performance. These ratings provide valuable insights into the likelihood of future performance limitations or lameness issues, guiding decisions regarding the purchase and management of the horse. The risks associated with orthopaedic lesions observed in thoroughbred yearling repository radiographs on future racing performance have been the focus of numerous studies (1–8). However, interobserver agreement risk for future racing performance and pre-purchase radiographically present lesions has received little attention. Only one study reports the agreement between observers for orthopaedic findings on pre-sale radiographs of thoroughbred yearlings, demonstrating an excellent agreement for the absence of lesions. There is a substantial variability for radiographic changes, particularly on what constitutes a pathologic change, particularly in regards to osteophytes and enthesophytes in the carpus and tarsus (9). This variability underscores the intricacies inherent in assessing radiographic findings.

A study by Jackson, et al. (9) engaged four specialist veterinary radiologists and revealed several factors influencing the agreement in radiographic interpretation, including lesion size, image quality, and observer experience. Larger or more pronounced findings typically showed higher levels of agreement, while smaller or subtle lesions led to reduced agreement among observers (8).

Radiographic evaluations are a cornerstone of the pre-purchase assessment for thoroughbred yearlings, providing critical information about potential future performance and health risks. However, there are notable gaps in current research, particularly concerning observer agreement and validating perceived risks against actual performance outcomes. Addressing these gaps through targeted research could enhance radiographic evaluations’ reliability and predictive value, ultimately improving decision-making processes in the thoroughbred yearling repository.

Despite the potential for substantial financial implications resulting from repository radiographic interpretation, few studies have assessed the agreement between veterinary surgeons in identifying pathology from yearling repository radiographs. The primary objective of the present study was to assess the level of agreement among interpreters for orthopaedic lesions identified in thoroughbred yearling repository radiographs using a pre-defined risk rating classification. We hypothesized that there would be a good agreement between observers for identifying lesions and a good to a fair agreement for their associated risk rating.

## Methods and materials

### Radiographic sets

Thirty sets of repository radiographs generated for any public auction through Magic Millions proprietary limited or William Inglis and Son Limited were retrieved from two private practice equine hospitals. Radiographic sets were selected randomly from a list of yearlings sold at auction between 2014 and 2018. The listed radiographic set was part of a larger study on thoroughbred yearling radiographic lesions and racing performance. Selection was performed by individuals blinded to the purpose of the study, with the selection distributed evenly between hospitals. All radiographic sets were comprised of the standard 36 radiographic views as mandated by the Australian thoroughbred yearling sales companies (Table 1) (10, 11).

**Table 1:** Overview of the standard radiographic views of repository radiographs.

Anatomic region	Views
Distal interphalangeal joint	Lateromedial
Metacarpophalangeal joint	Flexed lateromedial, Dorso 45–55° lateral-palmaromedial oblique elevated 5–10°, Dorso 45–55° medial-palmarolateral oblique elevated 5–10°, Dorsoproximal 30° -palmarodistal
Carpus	Flexed lateromedial, Dorso 50–60° lateral-palmaromedial oblique (DLPaMO), Dorso 70° medial-palmarolateral oblique (DMPaLO)
Metatarsophalangeal joint	Lateromedial, Dorso 45–55° lateral-plantaromedial oblique elevated 15° (DLPlMO), Dorso 45–55° medial-plantarolateral oblique dorsal 15°, Dorsoproximal 30° -plantarodistal
Tarsus	Lateromedial, Dorso 55–65° medial-plantarolateral oblique & Dorso 10–20° lateral-plantaromedial oblique
Stifle	Lateromedial (LM), Caudocranial, Caudolateral craniomedial oblique

### Radiographic reporting

A descriptive study was conducted using three highly experienced equine surgeons two American college of veterinary surgeons (ACVS) diplomates and one fellow of the Australian and New Zealand college of veterinary scientists (FANZCVS) with over a decade of experience, each in interpreting thoroughbred repository radiographs and performing a standardized evaluation of the pre-sales radiographs. Each observer utilized a list of orthopaedic lesions modified from Jackson, et al. (8) study to include more radiographic changes that may be observed during a repository examination, identifying lesions as present or absent. The list of modified radiographic changes was expanded to include more radiographic lesions observed in the repository, which are reported in the Supplementary Table S1.

The clinical significance of lesions to limit future race performance of flat racing thoroughbreds was further graded as low, moderate, and high-risk. Lesions considered to be low-risk include minor radiographic changes unlikely to affect future racing performance and cases with fragmentation where surgical removal has an excellent prognosis for future racing performance or, without removal, may delay the onset of training and/or shorten the length of future racing performance. Moderate-risk lesions will likely affect future race performance; if the lesion becomes clinical, treatment options are limited and will likely affect career longevity. Lesions considered to be high risk are likely to be unable to withstand the stress of training, and if they become clinical, the prognosis for return to racing is considered fair to poor.

### Categorization of lesions

The full list of radiographic lesions is reported in the Supplementary Table S1.

#### Distal interphalangeal joint

For observation, yearlings with modelling of the dorsal and dorsodistal aspect of the third phalanx were noted to have irregular bone formation or mineralization of the laminar attachments. Osteoarthritis changes in the joint included osseous alterations at the articular margins, resulting in surface irregularity, such as osteophyte or enthesophyte formation. Distal sesamoid sclerosis was characterized by increased bone radiopacity. Subchondral lucency (SCL) was defined as a focal radiolucency adjacent to the joint margin in the distal phalanx or as a well-demarcated lucency in the subchondral bone.

Distal phalangeal extensor process fragmentation was identified by focal osseous bodies detached from the parent bone. Rotation of the distal phalanx was described as any distal rotation away from the dorsal hoof wall. A negative palmar angle of the distal phalanx was observed when the palmar aspect of the distal phalanx was rotated below the distal aspect of the tip of the dorsal toe region.

#### Proximal interphalangeal joint

For analysis, yearlings were grouped based on the anatomic location or exposure to specific radiographic abnormalities of the pastern. Modelling of the dorsal aspect of the middle phalanx was defined as any irregular bone formation. Osteoarthritis was characterized by osseous changes at the articular margins of the joint, resulting in surface irregularity or the formation of osteophytes and enthesophytes. Osseous fragments were characterized as focal osseous bodies separated from a parent bone, occurring in the proximal interphalangeal joint (PIPJ). Subchondral lucency in the PIPJ was noted as a focal radiolucency adjacent to the joint margin or as a well-demarcated lucency in the subchondral bone in the proximal aspect of the middle phalanx or the distal aspect of the proximal phalanx.

#### Metacarpophalangeal and metatarsophalangeal joint

Radiographic lesions observed in the metacarpophalangeal and metatarsophalangeal joints included osseous fragments, characterized as focal osseous bodies separated from a parent bone, occurring within the joint margin from either the sagittal ridge, dorsoproximal, or proximopalmar locations. Subchondral lucency was defined as a focal radiolucency adjacent to the joint margin or as a well-demarcated lucency in the subchondral bone located in the proximal aspect of the proximal phalanx or the distal aspect of the third metacarpal or metatarsal bone. Osteoarthritis was identified by any osseous changes at the articular margins of the joint, resulting in surface irregularities, including osteophytes and enthesophytes. Supracondylar lysis (SCL) was assessed as local resorption of cortical bone of the third metatarsus or metacarpus proximal to the condyles underlying the palmar pouch of the joint capsule, as observed on the lateromedial radiograph.

**Figure 1:**
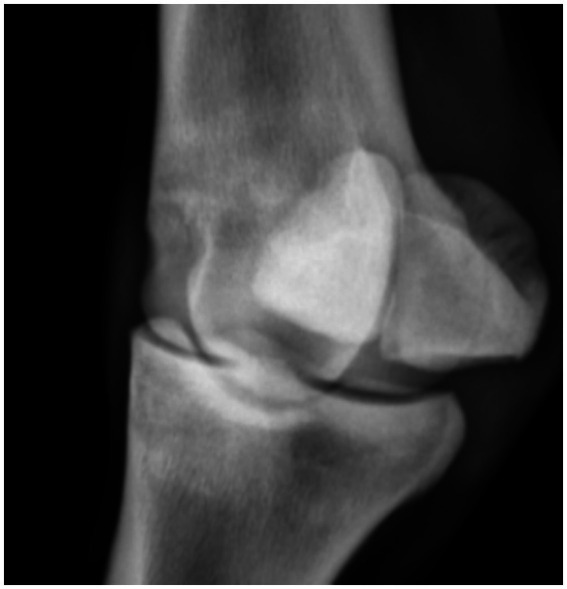
Sesamoiditis of the fore limb medial proximal sesamoid bone.

Sagittal ridge defects of the third metacarpal or metatarsal bone included focal contour irregularity of reabsorption or lysis without fragmentations. Fractures of the proximal sesamoid bones were reported based on their anatomical location. Modelling of the proximal sesamoid bone included lysis, irregular bone formation, sclerosis, and contour irregularity. Sesamoiditis was classified radiographically by irregular vascular channels greater than 2 mm in diameter with divergent or non-parallel borders in the proximal sesamoid bone, as depicted in Figure 1. Sesamoids were classified as elongated if the medial or lateral proximal sesamoids were 2 mm longer than the others on the same limb.

### Carpus

Radiographic lesions observed in the carpus included osseous cyst-like lesions (OCLLs), defined as focal, radiolucent areas that were either conical or spherical, located in the trabecular bone beneath the articular margin, specifically in the ulnar carpal bone. Osseous bodies separated from a parent bone were identified within the joint margin, occurring in the third, intermediate, radial, ulnar, and fourth carpal bones. Modelling of the carpal bones and distal radius included irregular bone formation, sclerosis, or lysis of the contour. Accessory carpal bone fractures encompassed all partial or complete breaks in the bone.

### Tarsus

Radiographic lesions observed in the tarsus included osteochondritis dissecans lesions, which were classified based on radiographic changes such as the presence of osseous fragments and corresponding areas of irregular radiolucency within the distal intermediate ridge of the tibia, the medial and lateral trochlear ridges of the tibia, and the medial and lateral malleolus of the tibia. Modelling of the dorsal proximal third metatarsal bone, third tarsal bone, and central tarsal bone was defined by any osseous changes occurring at the articular margins of the joint, resulting in surface irregularity. Slab fractures were considered bi-articular fractures of the central or third tarsal bones occurring on the dorsal plane. “Incomplete” slab fractures were uniarticular, non-displaced fractures, that have not become complete through the second articular surface. Osseous fragments were characterized as focal osseous bodies separated from a parent bone.

**Figure 2:**
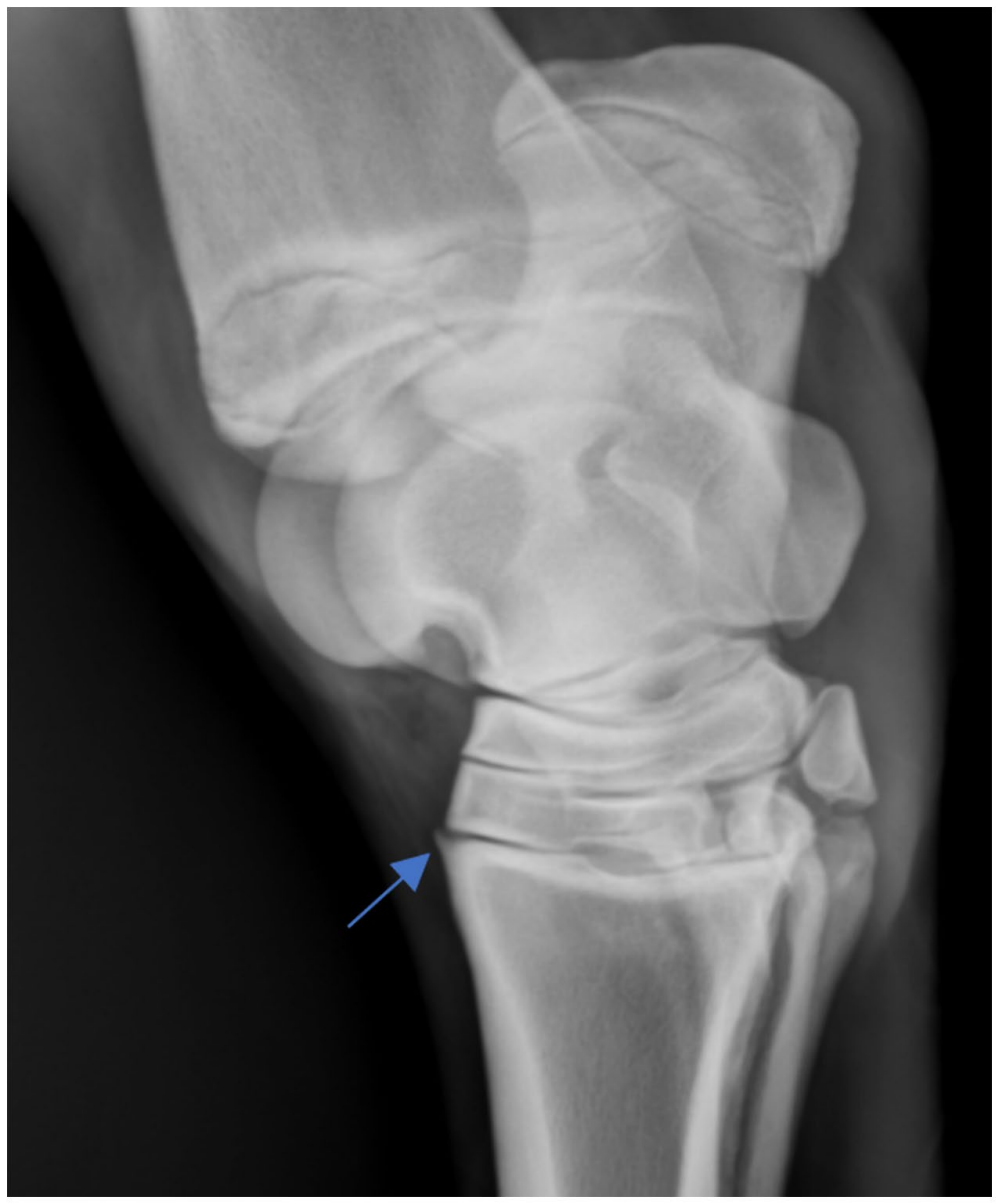
Osteophyte of the tarsometatarsal joint.

Osteophytes were defined as bony projections associated with the articular margins of the tarsometatarsal, proximal and distal intertarsal joints (Figure 2). Collapse of the central and third tarsal bones included joint ankylosis, narrowing, and compression. Wedging and convexity describe the dorsal border appearance of the central and third tarsal bones. Wedging involves either the central and third tarsal bones tarsal bones extending beyond the dorsal border of the third metatarsal bone, and convexity refers to the dorsal border curving plantar.

### Stifle

**Figure 3:**
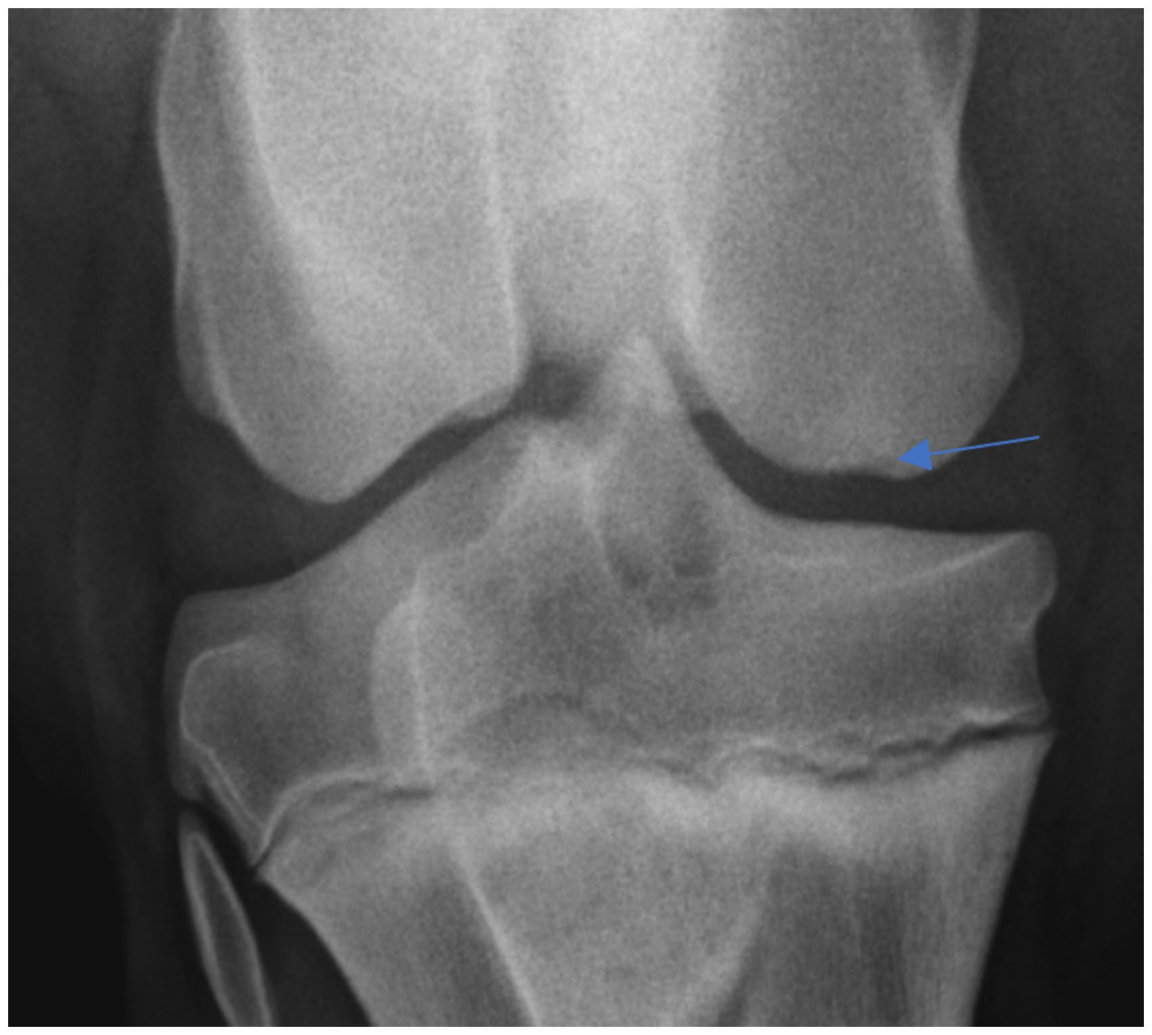
Medial femoral condyle shallow concave lucency.

Osteochondritis dissecans lesions were classified based on radiographic changes, including the presence of osseous fragments and corresponding areas of irregular radiolucency within the patella and the medial and lateral trochlear ridges of the femur. Subchondral lucency was defined as focal radiolucency beneath the bone surface, under the cartilage on the medial and lateral femoral condyles (Figure 3). Calcinosis circumscripta was identified as focal cutaneous mineralization located away from the articular margin. Radiographic changes in the meniscal ligaments included enthesophyte formation or osseous fragments originating from the parent bone. Radiographic osseous cyst-like lesions (OCLLs) were defined as focal, radiolucent areas that were either conical or spherical, located in the trabecular bone beneath the articular margin, either in the medial femoral condyle or proximal tibia.

Flattening of the distal articular contour of the medial femoral condyle was characterized by a loss of rounding or blunting of the condyle. The medial femoral condyle sclerosis was noted as increased bone radiopacity of the distal articular contour. Modelling of the extensor fossa, distal femoral condyles, and proximal tibia included any osseous irregular bone formation or surface irregularities. Fractures of the femoral condyle and tibial intercondylar eminence were focal osseous bodies detached from the parent bone.

### Data analysis

The percentage of agreement between observers was calculated with all yearlings, joints, and lesions grouped together but also independently by joint and lesion. The agreement was calculated in two ways. Firstly, radiographs were divided into three categories: agreement for lesion presence vs. agreement for lesion absence vs. disagreement, and then two categories with agreement (presence or absence) vs. disagreement. Categorical variables are reported using frequencies and percentages. Contingency tables were calculated for the relationship between 2 categorical variables. All analyses were performed using the R statistical software [R Core Team (2022). R: A language and environment for statistical computing. R Foundation for Statistical Computing, Vienna, Austria].

## Results

A total of 30 radiographic sets with 36 radiographic views were reviewed in each dataset (all yearlings, joints and lesions considered) rated by 3 observers for pathology and risk.

### Combined agreement per joint

Table 2 presents the combined agreement between all observers for all pathology considered for each joint. The proximal interphalangeal joint and distal interphalangeal joint had a near-perfect agreement for the absence of pathology, with observers agreeing 99.9 and 98.2% of the time, respectively. Observers have similar levels of agreement for the absence of pathology for the carpus (97.4%), stifle (96.8%), metacarpophalangeal joint (96%) and metatarsophalangeal joint (97.3%). The tarsus had the lowest level of agreement of all joints, with observers agreeing 94.2% of the time on the absence of pathology. The metacarpophalangeal joint and the tarsus had the highest level of disagreement for the presence of pathology, with observers disagreeing 3.5% of the time in the metacarpophalangeal joint and 5.2% of the time in the tarsus.

**Table 2:** Agreement between all three observers for combined pathologies by joint.

	Agreement of presence of pathology	Agreement of absence of pathology	Disagreement of pathology
Proximal interphalangeal joint	1 (0.1%)	719 (99.9%)	0 (0%)
Distal interphalangeal joint	0 (0%)	766 (98.2%)	14 (1.8%)
Carpus	5 (0.6%)	760 (97.4%)	15 (1.9%)
Metatarsophalangeal joint	7 (0.5%)	1,401 (97.3%)	32 (2.2%)
Stifle	4 (0.4%)	1,044 (96.8%)	31 (2.9%)
Metacarpophalangeal joint	6 (0.4%)	1,383 (96%)	51 (3.5%)
Tarsus	7 (0.5%)	1,244 (94.2%)	69 (5.2%)

### Agreement of specific pathology

The agreement between observers for specific pathology observed in the radiographic study by joint is reported in Table 3. The tarsus was the joint with the highest disagreement when there was pathology present. This was a result of observers disagreeing on the presence of osteophytes in the distal intertarsal joint (10%), proximal intertarsal joint (11.7%) and tarsometatarsal joint (35%). Observers disagreed on the presence of sesamoiditis in 28% of the yearlings and 16.7% of yearlings with sagittal ridge defects in the metacarpophalangeal joint.

**Table 3:** Agreement between observers for pathology by joint.

Pathology	Agreement	Disagreement
Distal interphalangeal joint		
Modelling toe of third phalanx	53 (88.3%)	7 (11.7%)
Carpus		
Modelling intermediate carpal bone	57 (95%)	3 (5%)
Modelling radial carpal bone	53 (88.3%)	7 (11.7%)
Metacarpophalangeal joint		
Proximal sesamoid focal lucency	57 (95%)	3 (5%)
Osteoarthritis of the metacarpophalangeal joint	56 (93.3%)	4 (6.7%)
Supracondylar lysis	56 (93.3%)	4 (6.7%)
Proximal sesamoid modelling	55 (91.7%)	5 (8.3%)
MC3 sagittal ridge defect	50 (83.3%)	10 (16.7%)
Sesamoditis	43 (71.7%)	17 (28.3%)
Metatarsophalangeal joint		
Proximal sesamoid modelling	57 (95%)	3 (5%)
Osteoarthritis of the metatarsophalangeal joint	57 (95%)	3 (5%)
MT3 sagittal ridge defect	56 (93.3%)	4 (6.7%)
Osseous fragment proximopalmar aspect first phalanx non-articular	55 (93.2%)	4 (6.8%)
Sesamoditis	54 (90%)	6 (10%)
Stifle		
Medial femoral condyle - sclerosis	57 (95%)	3 (5%)
OCD patella	59 (98.3%)	1 (1.7%)
OCD lateral trochlear ridge of the femur	55 (91.7%)	5 (8.3%)
Medial femoral condyle shallow concave lucency	53 (88.3%)	7 (11.7%)
Flattening of the medial femoral condyle	48 (80%)	12 (20%)
Tarsus		
OCD defect of lateral trochlear ridge talus	57 (95%)	3 (5%)
Modelling of the (3rd and central) tarsal bone	56 (93.3%)	4 (6.7%)
Lysis/sclerosis origin of the proximal suspensory	54 (90%)	6 (10%)
Osteophyte distal intertarsal joint	54 (90%)	6 (10%)
Osteophyte proximal intertarsal joint	53 (88.3%)	7 (11.7%)
Modelling dorsal proximal dorsal third metatarsal bone	45 (75%)	15 (25%)
Osteophyte tarsometatarsal joint	39 (65%)	21 (35%)

Observers had an excellent agreement for larger fragments with a 95% agreement for tarsal OCD defects of the lateral trochlear ridge, and patella OCD (98.3%). Additionally, there was an excellent agreement on osteoarthritis of the metacarpophalangeal joint (93.3%) and metatarsophalangeal joint (95%).

### Agreement on lesion risk

Table 4 reports the agreement between observers for risk per joint. Observers reached a consensus on risk for future racing performance for lesions 97.1% of yearlings. The proximal interphalangeal joint had a perfect level of agreement with 100% agreement followed by the distal interphalangeal joint at 98.2% agreement. Observers agreed on risk of tarsal pathology in 94.8% and metacarpophalangeal joint pathology 96.2% of the time. The agreement between observers for risk level is reported in Table 5.

**Table 4:** Combined agreement between observers for risk by joint.

	Agreement	Disagreement
Proximal interphalangeal joint	720 (100%)	0 (0%)
Distal interphalangeal joint	766 (98.2%)	14 (1.8%)
Carpus	764 (97.9%)	16 (2.1%)
Metatarsophalangeal joint	1,408 (97.8%)	32 (2.2%)
Stifle	1,048 (97.1%)	31 (2.9%)
Metacarpophalangeal joint	1,386 (96.2%)	54 (3.8%)
Tarsus	1,251 (94.8%)	69 (5.2%)

**Table 5:** Agreement between observers for risk by joint.

Joint	Agreement of no significate finding	Agreement of low-risk lesions	Agreement of moderate risk lesions	Disagreement
Proximal interphalangeal joint	719 (99.9%)	1 (0.1%)	0 (0%)	0 (0%)
Distal interphalangeal joint	768 (98.5%)	1 (0.1%)	0 (0%)	11 (1.4%)
Carpus	761 (97.6%)	6 (0.8%)	0 (0%)	13 (1.7%)
Metatarsophalangeal joint	1,404 (97.5%)	10 (0.7%)	0 (0%)	26 (1.8%)
Stifle	1,047 (97%)	10 (0.9%)	0 (0%)	22 (2%)
Metacarpophalangeal joint	1,389 (96.5%)	6 (0.4%)	3 (0.2%)	42 (2.9%)
Tarsus	1,259 (95.4%)	13 (1%)	0 (0%)	48 (3.6%)

## Discussion

The interpretation of pre-purchase radiographs remains a subject of ongoing debate among veterinary surgeons. A recent study (12) highlighted this issue, noting that a veterinary surgeons experience significantly affects the interpretation of radiographic lesions. Board-certified veterinary surgeons, in particular, showed lower concern for mild changes in the navicular bone and stifle. This may have contributed to the high level of agreement among observers regarding the absence of radiographic lesions across all joints in the current study. This finding aligns with previous research by Jackson, et al. (9), which reported a similar consensus among specialist veterinary radiologists.

Obtaining a comprehensive array of radiographic changes in a repository examination study can be challenging, especially in a randomly selected sample. More extensive or high-risk lesions may not be presented due to concerns about potential financial implications, such as a reduction in sale price. Additionally, acquiring a large sample size, particularly for the proximal interphalangeal joint region, is difficult. The reported prevalence of lesions in Australian Thoroughbred yearlings is around 6.3% (8), which contrasts with populations elsewhere, such as the USA, where only 1.3% (2) of yearlings display similar changes, and Japan, where the prevalence is as low as 0.2% (4). Consequently, the low sample size is representative of a typical repository examination.

Areas of disagreement in our study were notable in the presence of osteophyte formation in the tarsometatarsal joint and the modelling of the dorsal aspect of the third tarsal bone, with observers differing 35 and 25% of the time, respectively. These discrepancies may be attributed to variations in observer tolerance levels for osteophyte size. Similarly, Labens, et al. (13) found poor agreement in the assessment of the distal tarsal joint, suggesting that osteophytes may be unreliable indicators of osteoarthritis among observers. These findings indicate a need for more structured classification systems and standardized scales for evaluating specific radiographic lesions.

The study by Lacitignola, et al. (14) introduced a four-point grading scale for assessing osteophytosis in the metacarpo/metatarsophalangeal joint. Their findings indicated good intra-observer agreement among experienced veterinary surgeons, although slightly higher variability was observed among inexperienced observers. However, inter-observer correlation remained high, suggesting promising reliability of the grading system across different observers. The experienced observers demonstrated greater consistency in their assessments than inexperienced observers, who showed promising reliability. The current study utilized experienced observers, resulting in minimal disagreement regarding osteoarthritis of the metacarpo/metatarsophalangeal joint, with only 6.7% in the metacarpophalangeal and 5% in the metatarsophalangeal joint. Suggesting that experienced observers are adept at identifying and agreeing upon the presence of pathological changes in the metacarpo/metatarsophalangeal joint. Introducing a grading scale could prove beneficial in training inexperienced veterinary surgeons, as it provides a standardized framework for assessing and quantifying osteoarthritis.

Lesions characterized by a high degree of subjectivity, such as sclerosis and modelling in the proximal sesamoid bones, are difficult to objectively grade due to the presence of multiple differing classification scales in the literature (2, 15–18). In our study, observers recorded a notable 28.3% disagreement for sesamoiditis in the forelimbs and 10% in the hindlimbs. Additionally, disagreement reached 8.3% for the modelling of the proximal sesamoid bones. Jackson, et al. (9) additionally reported a fair to good intra-agreement observer and poor inter-agreement agreement for sesamoiditis, which is consistent with the findings of our study (9). The intricacies surrounding these lesions highlight the ongoing challenge of establishing a standardized approach to their evaluation and classification in radiographic interpretations.

Recently, Peat, et al. (19) introduced a novel grading scale to refine the reporting of radiographic alterations in the proximal sesamoid bones. Their proposed scale segregates vascular channel changes from abaxial irregular bone formation, offering an expanded assessment of proximal sesamoid pathology. A substantial level of agreement between the observers was reported, as indicated by a Cohen’s kappa coefficient of 0.65. This finding underscores the potential of the new grading scale to foster consensus among experienced observers in proximal sesamoid radiographic interpretation. However, it also suggests further investigation, particularly with less experienced observers, to fully assess the scale’s applicability and reliability across different expertise levels.

The tarsus and metacarpophalangeal joint exhibited the highest reported disagreement on risk, with observers disagreeing 5.2 and 3.8% of the time, respectively. This can be potentially attributed to the notable disagreements in the present study, particularly those involving the presence of osteophytes and sesamoiditis. A study by Lepeule, et al. (20) demonstrated good agreement (72%) for lesions of high severity but reported poorer agreement for mild to moderate lesions, a trend similarly observed in the metacarpo/metatarsophalangeal joint and carpus, aligning with our current findings. Brown, et al. (21) reported a similar trend when comparing the use of radiographs and cone beam computed tomography (CT) and the agreement of cervical articular process joint osteoarthritis. The observers had only slight to fair agreement for normal variation to mild osteoarthritis, while there was a moderate to substantial agreement for moderate to severe changes.

Numerous studies have documented that suboptimal radiographic quality and improper positioning can result in artifacts, concealed abnormalities, and misdiagnosis (9, 22, 23). It is possible that a significant source of disagreement with respect to the presence or absence of pathology may arise from suboptimal radiographic projections and variations in anatomical positioning. Variations in joint angles can unintentionally obscure certain lesions, such as defects in the sagittal ridge of the metacarpo/metatarsophalangeal joint and flattening of the medial femoral condyle. Observers exhibited a disagreement rate of 16% in the forelimbs and 6.7% in the hindlimbs with respect to defects in the sagittal ridge of the metacarpo/metatarsophalangeal joint in this study. The flexed lateromedial projection of the metacarpophalangeal and lateromedial metatarsophalangeal joint is the preferred projection for the identification of these defects; however, this projection can be commonly obliqued. The latter has been shown to be responsible for 14.9% of all radiographic errors and non-diagnostic views in the repository radiographs (9). The current study did not assess the radiographic positioning of each anatomical area to simulate the current repository conditions. While this may have contributed to some level of disagreement, it does represent the current repository scenario in which areas of disagreement arise between clinicians. Currently, there is no quality assurance in assessing the radiographic position or diagnostic quality of radiography presented for examination in the sale repository.

Multiple publications have sought to evaluate the risk of orthopaedic lesions for their ability to limit future racing performance (1, 7, 8, 24). In our investigation, we documented excellent observer agreement for the presence and risk of lesions (97.1%) across most joints among experienced veterinary surgeon. It is important to note that all observers involved in this research possessed specialist qualifications in equine surgery and extensive experience in interpreting pre-sale radiographs. Furthermore, the primary clinical focus of observers is thoroughbred horses, as the clientele served by veterinary surgeon can significantly influence the tolerance for radiographic changes. Recent research has shown that Western-performance horse veterinary surgeons tend to have a higher tolerance for radiographic changes in the tarsus compared to those specializing in English disciplines (12).

The use of experienced observers in our study is reflective of the current practices in the Australian thoroughbred sales industry, where equine surgeons typically interpret a significant percentage of sales radiographs. This suggests that there may be potential for establishing a standardized agreement on risk assessment before educating less experienced observers. However, including observers with less experience may diminish the overall agreement on risk. Recent studies have highlighted differences in the levels of concern for radiographic abnormalities between board-certified and non-board-certified practitioners (12). Currently, risk is established for performance-limiting lesions with a combination of evidence-based medicine based on previous studies for common changes and clinical experience with uncommon lesions.

A significant gap in the literature is the need for studies validating the perceived presumptive risk based on radiographic findings with actual performance outcomes. While many studies suggest potential risks, there is limited evidence directly correlating these presumptive risks with real-world racing results. Further research in this area could provide unique insights and enhance the validation of existing grading scales, ultimately improving the predictive accuracy of radiographic evaluations and informing better decision-making in the thoroughbred horse industry.

A limitation of our study was that there was no screening process for the 30 randomly selected radiographs to ensure diagnostic quality across all views. Given the paramount importance of high-quality radiographs for accurate interpretation, it would be beneficial to identify and address common errors that are prevalent in repositories. However, it could be argued that this is representative of sales conditions. Another notable limitation is the small number of lesions within the moderate to high-risk range, that were observed in each radiographic set. However, the occurrence of high-risk pathology presented to sale is low and as such this was a representative group.

A further limitation of the study is that the positioning of each joint was not considered in the analysis. Incorrect positioning can lead to underestimation of specific lesions or misdiagnosis. Reviews of repository radiographs have demonstrated that approximately 3.8% of radiographs presented to the repository are non-diagnostic. Of these non-diagnostic images, 14.9% were due to incorrect positioning of the flexed lateromedial view of the metacarpophalangeal joint. This incorrect positioning can result in misdiagnosis or disagreement between observers, impacting the reliability of the radiographic evaluations. The decision not to consider the positioning of each joint in the analysis was made to ensure the study’s representativeness of typical repository situations. Correct joint positioning is essential for accurate diagnosis and may have accounted for some disagreement in this study.

This study suggests that veterinary surgeons commonly agree on the absence of radiographic lesions but not necessarily on risk rating in yearling repository radiography. The utilization of a pre-defined risk rating system demonstrates agreement among experienced veterinary surgeon, warranting further research and possibly leading to the establishment of an industry-standard reporting and grading scale.

## Data Availability

The raw data supporting the conclusions of this article will be made available by the authors, without undue reservation.
