# Supplementary material for: Evaluation of agreement for radiographic lesions and risk for racing in thoroughbred yearling sale repository radiographs
Source: Front Vet Sci. 2024 Oct 3;11:1430993. doi: 10.3389/fvets.2024.1430993 (PMC11483323; doi:10.3389/fvets.2024.1430993)
Supplement: Supplementary file 1 [file Table_1.docx]

**Supplementary tables**

**Table one: List of radiographic lesions of the fore foot reported in Thoroughbred yearlings**

| Distal interphalangeal joint | Present | Absent |
| --- | --- | --- |
| Modelling dorsal aspect third phalanx |  |  |
| Modeling of dorsodistal aspect of distal phalanx |  |  |
| Distal interphalangeal joint modelling |  |  |
| Osteitis of the distal phalanx |  |  |
| Sclerosis of the distal sesamoid |  |  |
| Distal phalangeal extensor process fragment |  |  |
| Rotation of the third phalanx |  |  |
| Negative palmar angle of the third phalanx |  |  |
| Subchondral lucency distal phalanx |  |  |

**Table two: List of radiographic lesions of the pastern reported in Thoroughbred yearlings**

| Pastern | Present | Absent |
| --- | --- | --- |
| Modelling dorsal aspect middle phalanx |  |  |
| Osteophyte proximal interphalangeal joint |  |  |
| Middle phalanx proximal abaxial subchondral lucency |  |  |
| Middle phalanx proximal axial |  |  |
| Middle phalanx distal abaxial subchondral lucency |  |  |
| Middle phalanx distal axial subchondral lucency |  |  |
| Distal subchondral lucency abaxial proximal phalanx |  |  |
| Distal subchondral lucency axial proximal phalanx |  |  |
| Palmar osseous bodies middle phalanx |  |  |
| Dorsal osseous bodies middle phalanx |  |  |
| Distal osseous bodies proximal phalanx |  |  |

**Three: List of radiographic lesions of the fetlock reported in Thoroughbred yearlings**

| Metacarpo/metatarsophalangeal joint | Present | Absent |
| --- | --- | --- |
| Sagittal ridge osseous fragment third metacarpal/tarsus |  |  |
| Sagittal ridge defect of the third metacarpal/tarsus |  |  |
| Osseous fragment dorsoproximal aspect first phalanx |  |  |
| Osseous fragment proximopalmar aspect first phalanx articular |  |  |
| Osseous fragment proximopalmar aspect first phalanx non articular |  |  |
| Modelling of the proximal sesamoid bone |  |  |
| Sesamoiditis |  |  |
| Proximal sesamoid bone fracture – apical |  |  |
| Proximal sesamoid bone fracture – abaxial |  |  |
| Proximal sesamoid bone fracture – basal |  |  |
| Proximal sesamoid bone fracture – midbody |  |  |
| Sesamoid fracture bone – comminated |  |  |
| Supracondylar lysis |  |  |
| Proximal sesamoid elongation apical/ basal |  |  |
| Subchondral lucency abaxial proximal aspect of proximal phalanx |  |  |
| Subchondral lucency axial proximal aspect of proximal phalanx |  |  |
| Subchondral lucency abaxial distal aspect of third metacarpal/tarsus |  |  |
| Subchondral lucency axial distal aspect of third metacarpal/tarsus |  |  |
| Osteoarthritis metacarpo/metatarsophalangeal joint |  |  |

**Table four: List of radiographic lesions of the carpus reported in Thoroughbred yearlings**

| Carpus | Present | Absent |
| --- | --- | --- |
| Osseous cyst-like lesions ulnar carpal bone |  |  |
| Osseous fragment third carpal bone |  |  |
| Osseous fragment intermediate carpal bone |  |  |
| Osseous fragment radial carpal bone |  |  |
| Osseous fragment ulnar carpal bone |  |  |
| Osseous fragment fourth carpal bone |  |  |
| Modelling radial carpal bone |  |  |
| Modelling intermediate carpal bone |  |  |
| Modelling distal radius |  |  |
| Modelling third carpal bone |  |  |
| Accessory carpal bone osseous fragment |  |  |
| Accessory carpal bone fracture |  |  |

**Table five: List of radiographic lesions of the tarsus reported in Thoroughbred yearlings**

| Tarsus | Present | Absent |
| --- | --- | --- |
| Osteochondritis dissecans distal intermediate ridge tibia |  |  |
| Osteochondritis dissecans medial malleolus tibia |  |  |
| Osteochondritis dissecans lateral malleolus tibia |  |  |
| Osteochondritis dissecans lateral trochlear ridge talus |  |  |
| Osteochondritis dissecans medial trochlear ridge talus |  |  |
| Tarsometatarsal joint - osteophyte |  |  |
| Distal Intertarsal joint - osteophyte |  |  |
| Proximal tarsal joint - osteophyte |  |  |
| Wedging/convex appearance of the central and third tarsal bone |  |  |
| Collapsed appearance of the central and third tarsal bone |  |  |
| Osseous fragmentation of the dorsal aspect of the distal talus |  |  |
| Slab fracture of the third tarsal bone |  |  |
| Non-displaced slab fracture third tarsal bone |  |  |
| Displaced slab fracture of the third tarsal bone |  |  |
| Slab fracture of the central tarsal bone |  |  |
| Non-displaced slab fracture central tarsal bone |  |  |
| Displaced slab fracture of the central tarsal bone |  |  |
| Lysis/Sclerosis origin of the proximal suspensory |  |  |
| Modelling dorsal proximal dorsal third metatarsal bone |  |  |
| Modelling of the tarsal bones (third and central) |  |  |

**Table six: List of radiographic lesions of the stifle reported in Thoroughbred yearlings**

| Stifle | Present | Absent |
| --- | --- | --- |
| Osteochondritis dissecans medial trochlear ridge femur |  |  |
| Osteochondritis dissecans lateral trochlear ridge femur |  |  |
| Osteochondritis dissecans patella |  |  |
| Osseous cyst-like lesions medial femoral condyle |  |  |
| Flattening of the medial femoral condyle |  |  |
| Shallow subchondral lucency medial femoral condyle shallow |  |  |
| Sclerosis of the medial femoral condyle |  |  |
| Subchondral lucency lateral condyle femur |  |  |
| Subchondral lucency axial medial condyle |  |  |
| Subchondral lucency axial Lateral condyle |  |  |
| Modelling of the extensor fossa |  |  |
| Modelling distal femoral condyles |  |  |
| Modelling - proximal tibia |  |  |
| Avulsion fracture femoral condyle |  |  |
| Calcinosis circumscripta |  |  |
| Tibial intercondylar eminence fractures |  |  |
| Proximal tibia osseous cyst-like lesions |  |  |
